# Crisis-time efficiency in Eastern Poland's regional hospitals (2015–2024): a data envelopment analysis

**DOI:** 10.3389/frhs.2026.1715091

**Published:** 2026-02-05

**Authors:** Krystian Małyszko, Bartosz Pędziński, Dominik Maślach, Marcin Warpechowski, Ludmiła Marcinowicz

**Affiliations:** 1 Department of Health, Marshal’s Office of the Podlaskie Voivodeship, Bialystok, Poland; 2 Department of Developmental Age Medicine and Pediatric Nursing, Faculty of Health Sciences, Medical University of Bialystok, Bialystok, Poland; 3 Department of Public Health, Faculty of Health Sciences, Medical University of Bialystok, Białystok, Poland; 4 Diagnostic and Treatment Center, Lomza Medical Center Ltd., Lomza, Poland; 5 Department of Health, Podlaskie Voivodeship Office, Bialystok, Poland; 6 Department of Biostatistics and Medical Informatics, Medical University of Bialystok, Bialystok, Poland

**Keywords:** efficiency, data envelopment analysis (DEA), health system financing, crisis, COVID-19 pandemic, hospitals, health workforce, resource allocation

## Abstract

Hospitals in Poland's border regions face persistent staffing pressures and rising costs, and the COVID-19 pandemic further disrupted activity. We assessed year-by-year changes in operational and financial efficiency in three regional hospitals (BIA, LOM, SUW) in Podlaskie Voivodeship, on NATO's eastern flank, over 2015–2024. Input-oriented Data Envelopment Analysis with CCR (CRS) and BCC (VRS) models was applied in two domains (operational and financial), and 95% bootstrap confidence intervals were calculated for efficiency scores. Operational efficiency declined during the pandemic and partially recovered thereafter. Before COVID-19, mean TE_CCR ranged from 0.607 (95% CI: 0.571–0.643) in LOM to 0.909 (0.833–0.989) in SUW. In 2020–2021, TE_CCR fell to 0.746 (0.704–0.783) in BIA and 0.399 (0.371–0.410) in LOM, with SUW decreasing to 0.810 (0.731–0.870). Post-pandemic values showed partial rebound: 0.858 (0.781–0.946) in BIA, 0.602 (0.565–0.634) in LOM, and 0.830 (0.758–0.913) in SUW. For LOM, operational TE_CCR dropped as low as 0.399 while financial TE_CCR remained at or above 0.94, illustrating a marked divergence between service delivery and financial performance. Across periods, PTE_BCC remained high, indicating scale efficiency as an important source of inefficiency. Financial efficiency showed a similar trough and recovery. Pandemic-period TE_CCR declined to 0.785 (0.766–0.798) in BIA and 0.951 (0.925–0.967) in SUW, while LOM remained relatively stable at 0.960 (0.947–0.970). Post-pandemic values increased to 0.928 (0.863–0.959) in BIA and 0.949 (0.901–0.979) in SUW, with LOM at 0.940 (0.919–0.958). Several confidence intervals did not include 1.00, indicating persistent inefficiency components. Overall, the decade shows a distinct pandemic-related dip followed by partial normalisation, with between-hospital heterogeneity and recurrent scale-related shortfalls in both domains. These results support routine, domain-specific efficiency monitoring as a tool for transparent performance tracking in strategically sensitive border regions. However, findings are constrained by the very small three-hospital sample, low discriminatory power of annual frontiers and the use of aggregated administrative data. Key methodological constraints are summarised in the Limitations section and should be considered when interpreting the findings.

## Introduction

Poland's healthcare system is based on universal and mandatory health insurance, with the National Health Fund (NFZ) acting as the single purchaser. Hospital care is predominantly financed via lump-sum contracts under the Basic Hospital Healthcare Security System (PSZ), complemented by targeted grants and task-specific subsidies in exceptional situations (1). While this architecture provides budgetary stability, it does not always reward efficiency or flexible responses to changing needs (2).

According to Statistics Poland (GUS), compiled under the System of Health Accounts (SHA 2011), public health expenditure rose from PLN 80.3 bn in 2015 to PLN 229.1 bn in 2024 (4.4% and 6.3% of GDP, respectively) (3). In the analysed provincial hospitals, employment-related expenses (salaried and civil-law contracts) accounted for ∼55% of operating costs in 2015 and exceeded 73% in 2024 (authors' calculations from audited statements, 2015–2024). Workforce shortages, wage pressure, and the expansion of flexible employment forms underpin this trend, and ageing of staff reinforces it: between 2012 and 2022, the share of physicians aged ≥65 years rose from 20.4% to 25.7%, and that of nurses from 5.4% to 22.3% (4).

The COVID-19 pandemic exposed the limits of rigid organisational structures and lump-sum financing; hospitals reorganised care under resource constraints and staffing shortages. The literature emphasises rapid decision-making, adaptation of procedures, redistribution of staff, and strengthened communication (5–7). The regional setting also matters: Podlaskie Voivodeship borders Belarus, Lithuania, and the Kaliningrad exclave, placing it on NATO's eastern flank and increasing the strategic salience of local hospital capacity during pandemics and potential geopolitical or migration crises (8). In such a borderland context, deviations from strict efficiency can also be read as an indication of potential reserve capacity in beds and staff that could be mobilised during sudden shocks, such as pandemics or migration and security crises. In this study, we therefore treat efficiency analysis not only as a way to identify waste, but also as a way to approximate the latent capacity of regional hospitals to respond to future crises (9, 10). Policy analyses, although not focused specifically on pandemics, suggest that payment frameworks which integrate efficiency and quality while accommodating complexity and provider heterogeneity perform better than one-size-fits-all tariffs (11). Although conducted outside Poland's borderland context, this work informs our choice of period stratification and guides interpretation of efficiency changes during crisis and non-crisis years.

Against this background, Data Envelopment Analysis (DEA) provides a non-parametric benchmark of relative efficiency using multiple inputs and outputs without a specified production function (12). It is widely applied in hospital research across regions and income levels, including pandemic-period assessments that show decline-and-rebound patterns (13–16). In Poland, however, DEA has mainly been applied at the voivodeship (regional) level, limiting managerial usefulness for individual hospital governance (17, 18). This study extends the evidence by providing a ten-year, hospital-level panel for three provincial providers in Podlaskie Voivodeship and by reporting both operational and financial efficiency with period summaries.

**Study hypotheses.** Guided by the context above and prior evidence, we test the following hypotheses:

H1 (period dynamics). Relative efficiency declined during the COVID-19 period (2020–2021) and partially rebounded after 2022 in at least one domain (operational or financial).
H2 (scale vs. technique). In hospital-years where PTE_BCC ≈ 1.00 but TE_CCR < 1.00, the shortfall is chiefly due to scale mismatch (scale efficiency <1.00) rather than pure technical inefficiency.
H3 (between-hospital heterogeneity). Efficiency trajectories differ by hospital, with SUW closer to the frontier on average, while BIA and LOM show greater year-to-year variability.
H4 (cross-domain relation). Year-to-year patterns in operational and financial scale efficiency are only weakly aligned, allowing hospitals to perform well in one domain while lagging in the other.

## Materials and methods

### Study design and setting

We conducted a retrospective panel study of three public regional hospitals in Podlaskie Voivodeship, north-eastern Poland, covering the years 2015–2024. Efficiency frontiers were estimated separately for each calendar year, so that all peer comparisons are strictly within-year. For reporting clarity, annual results are summarised into three periods: pre-pandemic (2015–2019), pandemic (2020–2021), and post-pandemic (2022–2024). This period stratification is presentational only, does not alter the underlying DEA specification, and is consistent with earlier hospital efficiency studies using DEA and related distance-function approaches (13, 19–20).

### Hospitals (decision-making units) and regional context

The analysis covered the Provincial Integrated Hospital in Bialystok (BIA), the Provincial Hospital in Lomza (LOM), and the Provincial Hospital in Suwalki (SUW), which provide core inpatient specialist care for approximately 1.17 million residents of Podlaskie Voivodeship. Based on complete, audited annual data, mean inpatient volumes were ∼27,000 at BIA (59,398 in 2024), ∼17,000 at LOM, and ∼35,000 at SUW. Each hospital-year constituted a decision-making unit (DMU), yielding 30 DMUs (3 hospitals × 10 years).

All three institutions are financed by the National Health Fund (NFZ) and belong to the Basic Hospital Healthcare Security System (Polish: System Podstawowego Szpitalnego Zabezpieczenia Świadczeń Opieki Zdrowotnej, PSZ), providing a comparable core of inpatient specialist services within this network. Differences in scale (bed stock, activity volume) are therefore treated as endogenous to frontier construction rather than as grounds for exclusion. Year-wise benchmarking limits the influence of system-level reforms and macro trends on peer comparisons. According to the NFZ classification (accessed 24 September 2025), BIA, LOM, and SUW are the only PSZ network hospitals in Podlaskie at levels II–III; they account for the majority of regional admissions and surgical procedures and are located centrally (Bialystok), in the west (Lomza), and in the north-east (Suwalki), respectively.

### Variables and indicators

We estimated two complementary DEA models to reflect distinct managerial perspectives and to avoid mixing heterogeneous objectives within a single frontier (21–23).

### Operational (technical) efficiency model

Inputs (4): number of beds; number of physicians; number of nurses/midwives; number of other medical staff.

Outputs (2): number of hospitalisations; number of surgical procedures.

These inputs represent controllable capacity and skill-mix at the hospital level, while the outputs capture core service production under resource and staffing constraints. Similar input–output sets have been used in hospital DEA studies focusing on technical efficiency and service throughput (21–23).

### Financial efficiency model

Inputs (2): total operating costs; number of beds.

Outputs (3): net revenue from services; number of hospitalisations; number of surgical procedures.

Here, inputs measure resource absorption, whereas outputs jointly reflect financial performance and activity throughput under prevailing payment arrangements. Using beds as an input in both models allows consistent treatment of physical capacity while distinguishing technical and financial perspectives (21–23).

### Operational definitions

Annual counts of physicians, nurses/midwives, and other medical staff aggregate salaried and civil-law arrangements at the institutional level without double counting. Bed numbers reflect the reported inventory for each year. Hospitalisations and surgical procedures are annual volumes consistent with reporting to the Marshal's Office of the Podlaskie Voivodeship.

### Contextual (non-DEA) indicators

For descriptive context, we report average length of stay (LOS), throughput (annual patients per bed), and average bed occupancy (mean bed utilisation). These indicators inform interpretation only and are not DEA variables. Financial descriptors include personnel cost per physician and per nurse/midwife (total annual remuneration, including civil-law contracts, divided by average annual headcount).

All variables satisfy DEA monotonicity (non-negative values; expanding inputs does not reduce the feasible output set; higher outputs indicate better performance). An input orientation was adopted to reflect greater short-run managerial control over staffing, bed stock, and spending than over demand and case-mix. The total of 30 DMUs satisfies the commonly used rule.

*n* ≥ max[m × s, 3(m + s)] for both models (operational: m = 4, s = 2 → threshold 18; financial: m = 2, s = 3 → threshold 15).

### DEA specification and estimation

We estimated yearly, input-oriented envelopment models under CCR (constant returns to scale, CRS) and BCC (variable returns to scale, VRS). We report TE_CCR (total technical efficiency under CRS), PTE_BCC (pure technical efficiency under VRS), and scale efficiency (SE) defined as SE = TE_CCR/PTE_BCC. Frontiers were computed separately for each calendar year so that hospitals are compared only within the same year. Period summaries aggregate yearly scores for presentation and do not imply a pooled multi-year frontier. Comparable DEA-based assessments of hospital efficiency, productivity, and reform or crisis impacts have been reported in diverse health systems (24–28).

Scale effects were interpreted through SE. Cases with PTE_BCC ≈ 1.00 and TE_CCR < 1.00 were interpreted as scale-related shortfalls, indicating that hospitals operated at a locally inefficient scale despite near-frontier pure technique. Returns-to-scale (RTS) classifications—CRS, decreasing returns to scale (DRS), and increasing returns to scale (IRS)—were derived for the operational model based on the BCC specification and used descriptively in the Results. Under CRS, proportional changes in inputs lead to proportional changes in outputs; DRS indicate that expanding scale yields less-than-proportional output gains, while IRS indicate the opposite. RTS diagnostics were not produced for the financial model, as the cost-based input structure and small number of DMUs per year make scale labels harder to interpret in that domain.

### Statistical analysis and bootstrap DEA

To quantify uncertainty around deterministic DEA scores, we applied a non-parametric, bias-corrected bootstrap following Simar and Wilson (29). Bootstrap resampling was implemented separately for each calendar year and for both variable sets (operational and financial), treating each hospital-year as a DMU. Resampling with replacement was performed at the DMU level, preserving the joint input–output structure.

For each hospital-year, we obtained bias-corrected TE_CCR and PTE_BCC estimates and 95% percentile bootstrap confidence intervals. Uncertainty was thus quantified for the CRS- and VRS-based efficiency scores. Scale efficiency (SE) was computed as the ratio of bias-corrected TE_CCR to bias-corrected PTE_BCC for each hospital-year; because SE is a deterministic transformation of these two scores, no separate confidence intervals were derived for SE. We used 2,000 replications with *α* = 0.05. Details on bootstrap implementation (including random seeds) are available from the authors on request.

### Second-stage (truncated) regression

To explore whether contextual covariates were associated with relative hospital (in) efficiency, we estimated exploratory second-stage models using truncated regression with left truncation at 0. Hospital-year inefficiency was defined as 1−*θ*, where *θ* denotes the DEA-based efficiency score, and served as the dependent variable in all specifications.

For the operational model, regressors were average bed occupancy (capacity utilisation), average LOS, and the share of physicians on civil-law contracts; period dummies identified COVID (2020–2021) and post-COVID (2022–2024) years. For the financial model, covariates included the number of beds (size proxy), bed occupancy, LOS, the share of physicians on civil-law contracts (cost structure), the share of surgical cases in total hospitalisations (case-mix proxy), and the same period dummies. Standard errors and *p*-values were obtained from maximum-likelihood estimation of truncated normal models.

### Data sources

Operational data (staffing, bed capacity, inpatient volumes, procedures) were obtained from the Marshal's Office of the Podlaskie Voivodeship under the Polish Access to Public Information Act. Financial data (revenues, personnel costs, and other operating expenses) were extracted from each hospital's Public Information Bulletin (BIP) with audited annual statements. Only complete and verified data were used.

### Data handling and reporting

**Units and scaling.** Financial variables were analysed in nominal PLN, and DEA was computed on original-level values without deflation or rescaling. Because benchmarking is within-year, common price changes do not affect comparability across DMUs in a given year.

**Quality control.** We performed double data entry and verification, followed by independent re-computation using a spreadsheet-based solver to confirm numerical stability of DEA scores. Extreme values were re-verified in the source documents but not trimmed, given the small number of peers per year.

**Missing data.** No missing data were identified across years or variables; imputation was therefore not required.

**Reporting precision.** DEA scores are reported to four decimal places. Period aggregates are arithmetic means of the constituent yearly values for each hospital and model.

### Scope of inference

DEA benchmarks relative efficiency rather than absolute performance. Contextual indicators and cross-domain summaries (operational vs. financial) are used to aid interpretation but are not modelled as causal drivers. The second-stage truncated regressions are exploratory and intended to complement, not replace, the primary DEA benchmarking.

### Software and reproducibility

All analyses were performed in R version 4.5.1 using the Benchmarking package version 0.33 (CRAN, 18 February 2025) (30). DEA estimation relied on the dea() function with input orientation. Bootstrap procedures and plotting scripts are available from the corresponding author upon reasonable request; data access and code availability are also summarised in the Data Availability statement.

### Ethics

The study used only aggregated operational and financial data at hospital level, obtained from routine reporting systems of the Marshal's Office of the Podlaskie Voivodeship and from hospitals' Public Information Bulletins (Biuletyn Informacji Publicznej, BIP) in response to formal requests under the Polish Access to Public Information Act. The dataset contained no individual patient or staff records and no personal data within the meaning of applicable data-protection law. According to Polish law, secondary analyses based solely on such non-identifiable aggregated administrative data are exempt from review by a bioethics committee; therefore, formal ethics approval and individual informed consent were not required for this study.

### Future methodological extensions

Because the panel consisted of only 30 DMUs (3 hospitals over 10 years), Malmquist or window DEA would likely yield unstable frontiers. We therefore relied on bootstrap DEA and second-stage regression as our main robustness tools and recommend Malmquist analysis for future studies with larger panels.

## Results

### Narrative results

**Figure 1:**
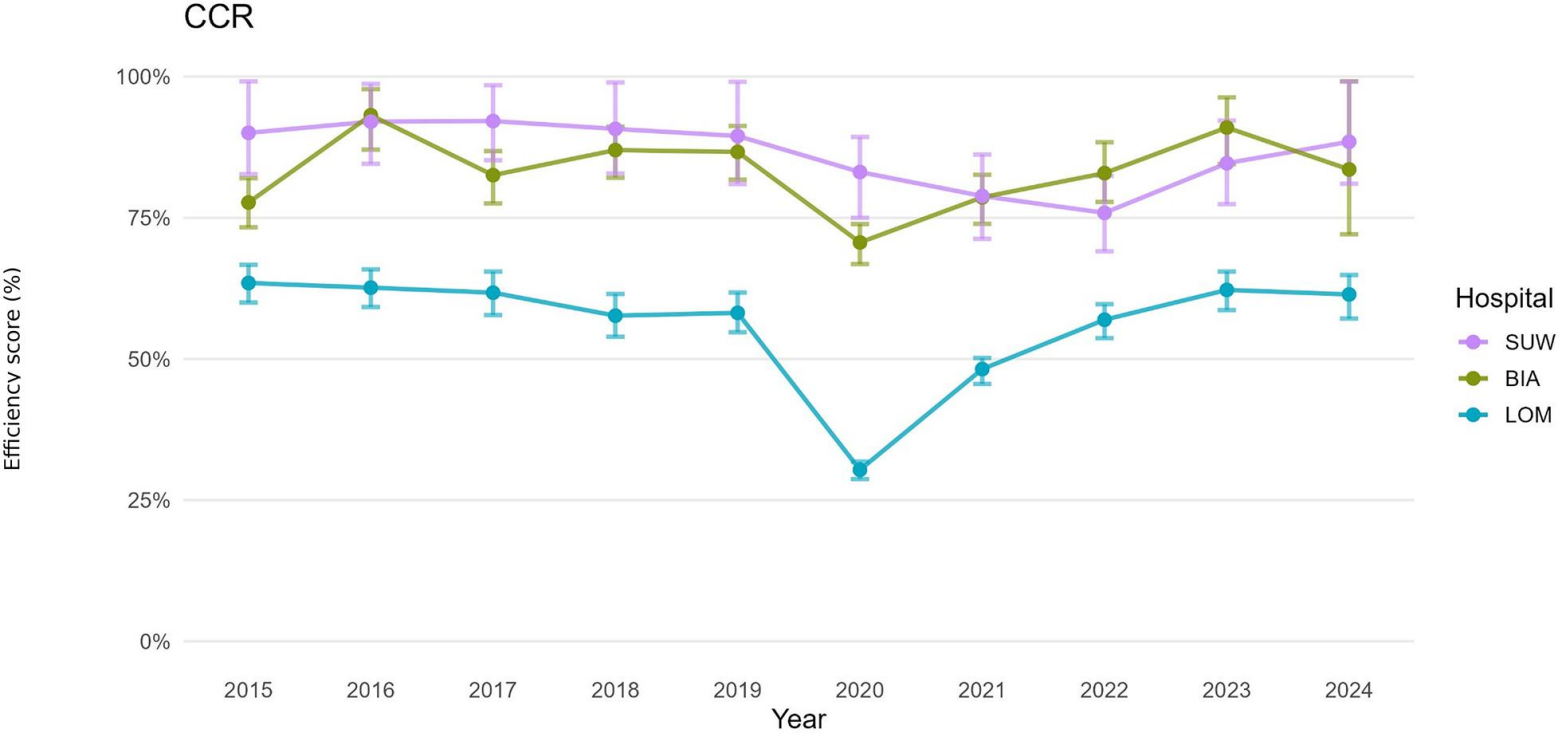
Hospital efficiency over time (CCR DEA model)—operational set of variables.

**Figure 2:**
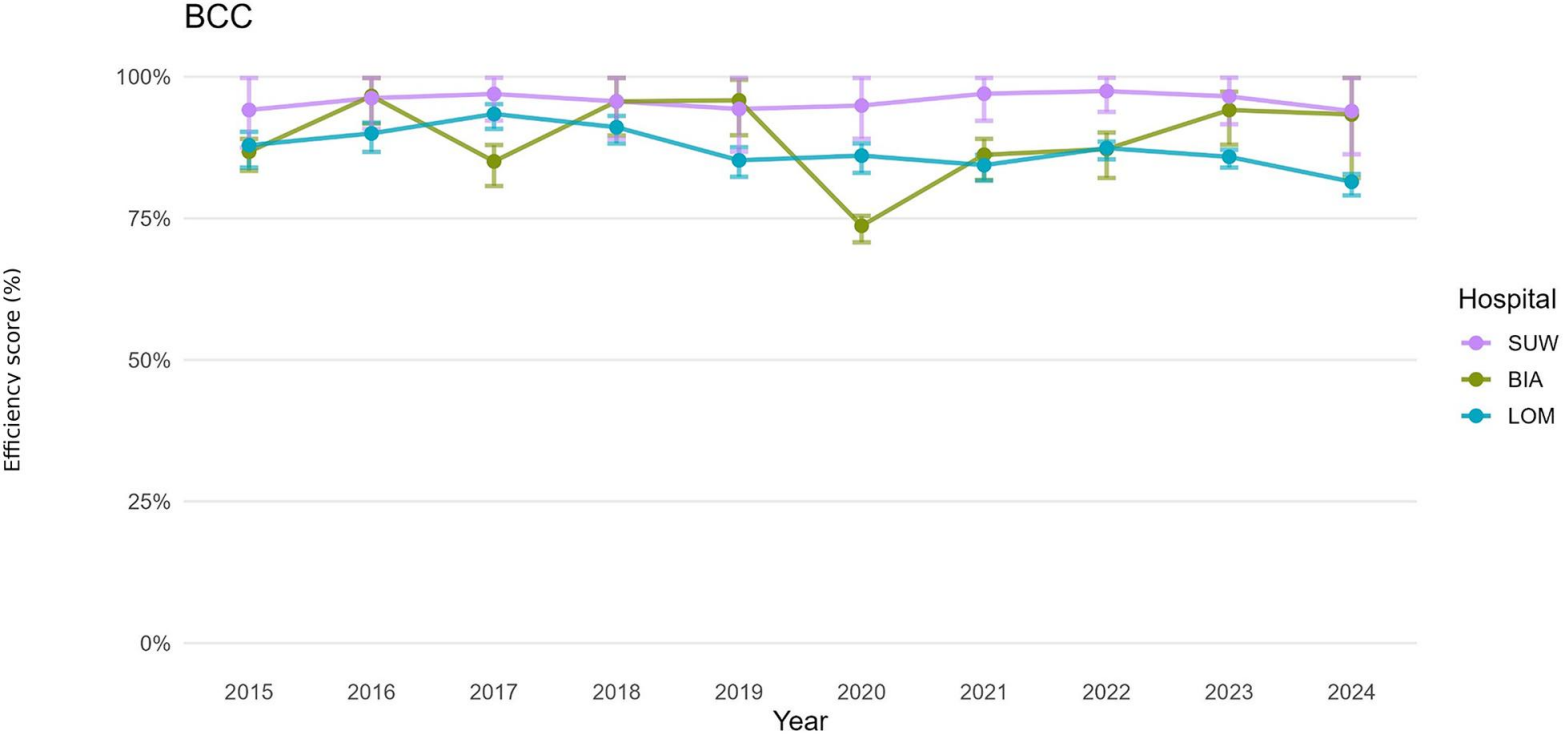
Hospital efficiency over time (BCC DEA model)—operational set of variables.

**Figure 3:**
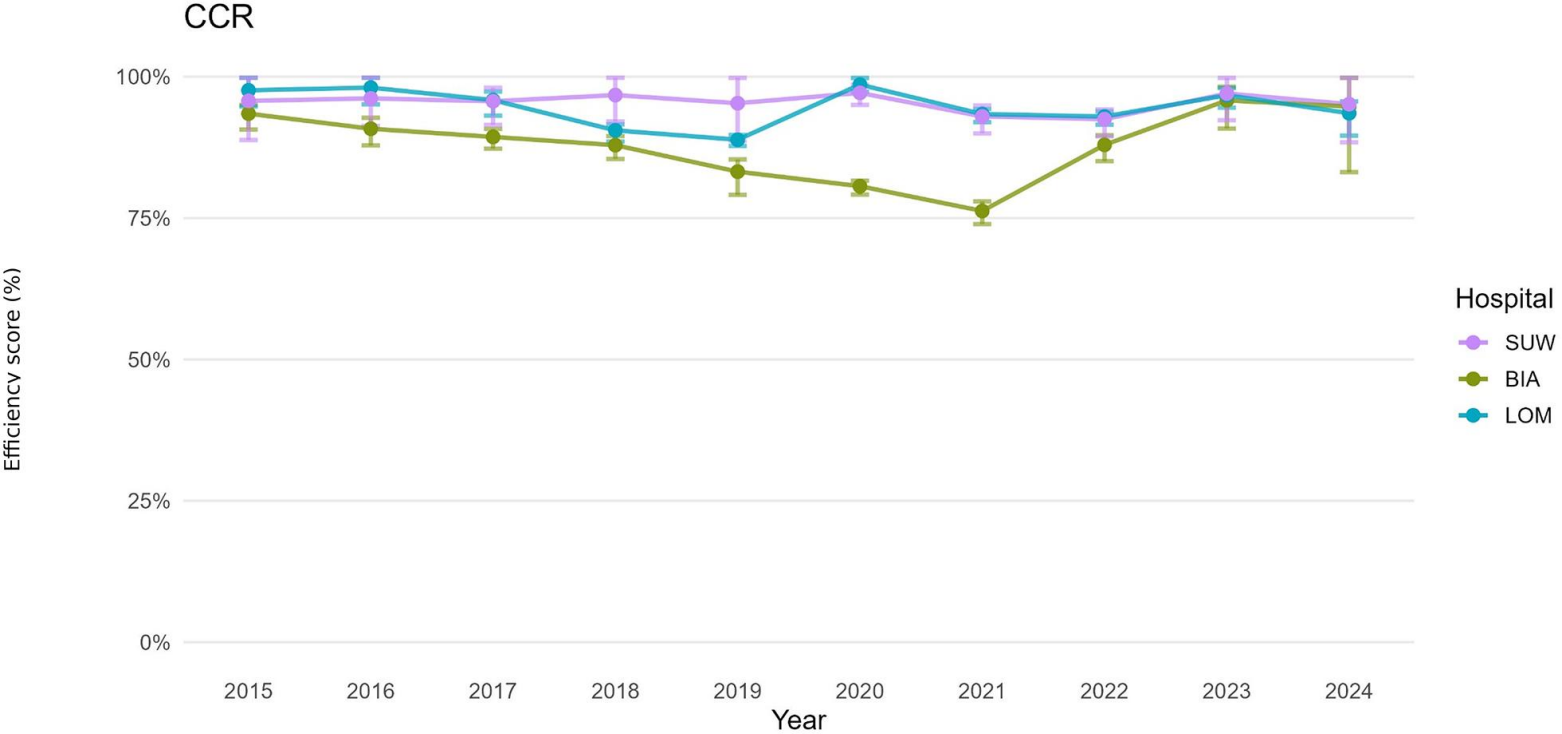
Hospital efficiency over time (CCR DEA model)—financial set of variables.

**Figure 4:**
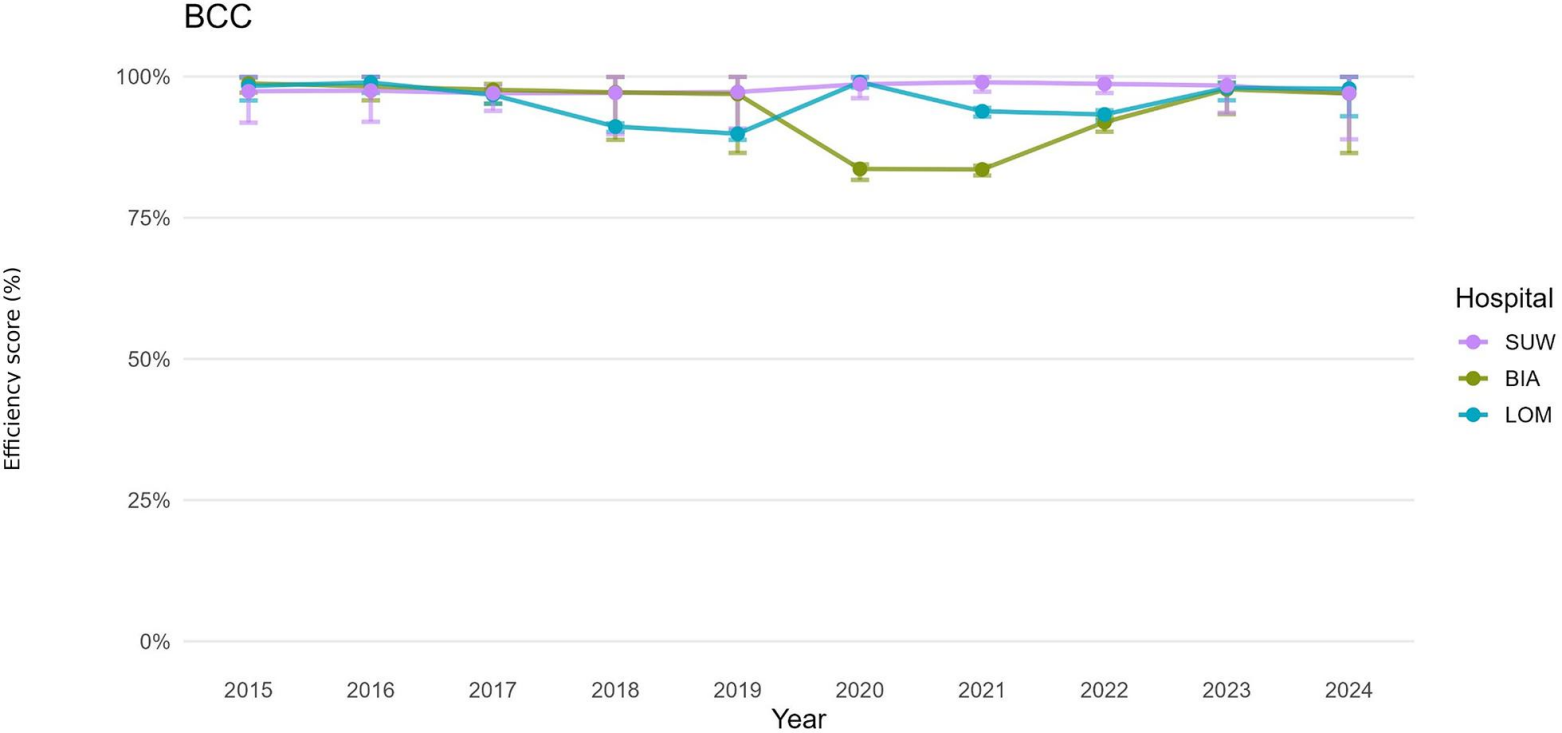
Hospital efficiency over time (BCC DEA model)—financial set of variables.

**Figure 5:**
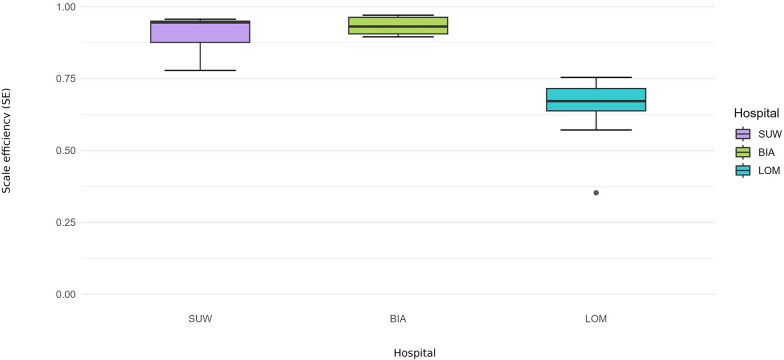
Scale efficiency (SE) 2015–2024—operational set.

**Figure 6:**
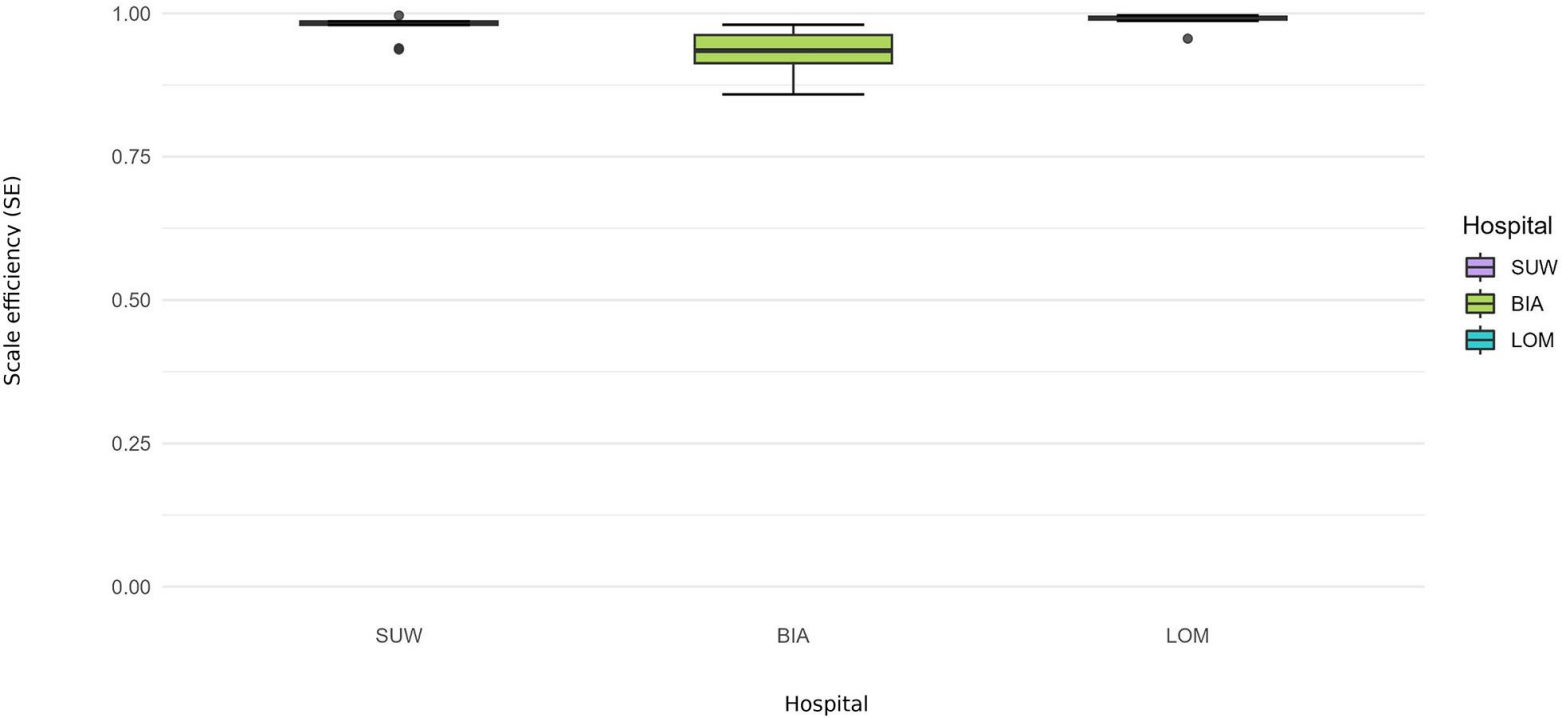
Scale efficiency (SE) 2015–2024—financial set.

For both the operational and financial domains, we defined hospital-year decision-making units (DMUs). For each DMU, we estimated bias-corrected efficiency under the CCR (constant returns to scale, CRS) and BCC (variable returns to scale, VRS) models, together with 95% confidence intervals obtained by bootstrap resampling (Table 3). For presentation, annual trajectories are summarised by period: pre-pandemic (2015–2019), pandemic (2020–2021), and post-pandemic (2022–2024). Figures 1–6 display year-by-year profiles, Tables 1, 2 provide context for interpreting the DEA results, and Table 3 reports period means with 95% CIs.

**Table 1:** Changes in employment, bed capacity and key operational indicators in three regional hospitals of Podlaskie Voivodeship, Poland.

Indicator	Hospital name	2015	2016	2017	2018	2019	2020	2021	2022	2023	2024	Percent Change 2024 vs. 2015
Physicians—employed	BIA	119	113	98	99	99	100	112	108	112	112	−6%
	LOM	40	36	30	30	31	31	33	37	42	40	2%
	SUW	78	73	70	73	65	60	56	69	66	58	−26%
Physicians—contractors	BIA	62	46	92	121	114	185	221	105	96	80	29%
	LOM	149	145	139	139	157	157	180	188	182	179	20%
	SUW	97	133	141	96	95	90	106	158	164	122	26%
Nurses and midwives—employed	BIA	485	489	483	488	496	528	533	506	520	505	4%
	LOM	456	465	470	474	478	454	455	468	482	557	22%
	SUW	382	400	415	425	422	421	411	413	424	425	11%
Nurses and midwives—contractors	BIA	46	58	106	121	114	113	102	78	69	52	13%
	LOM	0	0	0	20	26	30	36	34	35	40	-
	SUW	15	16	19	48	68	68	90	99	91	122	713%
Other medical staff	BIA	456	479	498	490	522	518	466	577	592	571	25%
	LOM	450	452	605	632	643	623	642	662	662	675	50%
	SUW	268	287	291	311	315	322	345	332	344	289	8%
Number of beds	BIA	641	641	641	642	609	617	617	536	494	503	−22%
	LOM	632	626	626	631	496	528	509	489	495	506	−20%
	SUW	448	448	456	456	423	423	418	414	417	417	−7%
Acute beds	BIA	20	20	25	25	25	25	30	25	25	25	25%
	LOM	9	12	12	12	10	10	15	11	11	11	22%
	SUW	17	17	17	17	17	17	17	17	17	17	0%
Number of treated	BIA	24,505	25,528	25,306	24,068	23,297	18,944	21,207	20,126	22,636	59,398	142%
	LOM	21,562	21,688	21,995	20,499	19,803	9,876	13,133	17,247	19,212	15,522	−28%
	SUW	34,545	35,186	34,948	36,201	35,686	28,958	32,212	33,778	37,617	38,541	12%
Average length of stay	BIA	6.5	5.8	5.7	6.0	5.5	6.0	5.8	5.7	5.1	8.2	27%
	LOM	5.4	5.2	5.1	6.6	4.9	5.3	5.1	4.3	4.0	5.1	−5%
	SUW	3.2	3.2	3.0	2.8	2.8	2.9	2.9	2.6	2.3	2.6	−19%
Throughput rate	BIA	38	40	39	37	38	31	34	38	46	118	209%
	LOM	34	35	35	32	40	19	26	35	39	31	−10%
	SUW	77	79	77	79	84	68	77	82	90	92	20%
% average bed occupancy rate	BIA	67	68	67	62	62	53	60	65	71	88	32%
	LOM	62	62	63	58	68	34	44	51	56	57	−8%
	SUW	68	68	63	61	65	55	60	59	56	67	−1%

**Table 2:** Financial performance and operating costs (thousand PLN) of three regional hospitals in Podlaskie Voivodeship, Poland.

Indicator	Hospital name	2015	2016	2017	2018	2019	2020	2021	2022	2023	2024	Percent Change 2024 vs. 2015
Net revenue from the sale of services.	BIA	105,910	112,168	124,173	138,442	154,296	166,863	194,042	217,710	260,233	287,530	146%
	LOM	102,964	109,798	112,705	124,560	132,750	164,419	197,541	198,659	243,113	274,364	136%
	SUW	82,766	91,321	97,525	113,904	133,414	144,622	176,718	182,443	224,366	257,381	171%
Operating expenses	BIA	117,495	127,951	143,616	167,304	193,314	212,977	261,916	270,621	306,237	340,415	161%
	LOM	102,819	109,868	116,136	137,755	152,746	168,759	217,882	220,433	260,002	302,638	153%
	SUW	90,120	96,586	106,285	125,037	139,795	152,921	195,247	203,144	236,056	273,677	162%
Amortization	BIA	7,521	9,750	10,776	10,949	15,612	16,982	16,964	18,488	18,415	13,589	145%
	LOM	8,252	8,197	7,943	7,615	9,156	10,950	11,615	10,225	11,051	11,786	34%
	SUW	8,260	7,469	6,669	6,349	7,842	9,980	10,341	11,738	11,373	10,612	38%
Materials and energy	BIA	25,079	27,957	30,534	31,564	34,313	36,608	42,451	43,576	49,150	49,184	96%
	LOM	23,530	25,258	26,969	28,148	28,783	27,156	41,430	41,211	48,023	52,946	104%
	SUW	18,388	19,659	22,533	26,581	29,620	33,717	41,258	41,966	51,638	61 826	181%
External services (excluding contracts)	BIA	30,578	32,925	37,258	47,040	54,241	59,158	68,407	64,817	70,121	85,868	129%
	LOM	25,704	27,549	25,295	32,466	34,416	42,441	53,983	55,681	67,978	81,618	164%
	SUW	20,057	22,079	23,781	27,863	31,245	36,864	49,951	52,755	65,148	80,135	225%
Salaries and wages with benefits	BIA	53,010	56,050	63,891	76,501	87,909	98,758	132,463	141,874	166,302	189,293	214%
	LOM	43,328	46,541	52,438	64,591	75,567	86,366	108,864	110,856	130,196	153,178	200%
	SUW	39,583	43,309	48,710	58,773	67,063	71,152	92,464	95,586	106,709	119,833	170%
Salaries from civil law contracts	BIA	17,153	19,158	23,671	31,243	36,299	38,175	48,563	43,782	46,919	85,868	174%
	LOM	18,454	20,035	20,742	27,064	28,010	33,541	45,333	46,699	57,432	81,618	211%
	SUW	8,564	9,528	10,210	13,352	15,240	20,263	29,745	28,305	35,718	80,135	317%
Other operating revenue	BIA	8,076	10,817	11,490	11,462	17,828	34,591	50,797	26,529	20,435	15,889	153%
	LOM	757	3,480	6,848	3,987	2,601	6,872	8,681	11,417	3,912	4,336	417%
	SUW	2,801	1,586	2,277	3,398	2,672	5,040	4,945	4,139	2,971	8,389	6%
Grants	BIA	5,383	5,961	6,981	9,188	10,911	12,341	13,655	18,694	21,671	25,562	303%
	LOM	6,233	5,667	4,881	5,331	6,846	6,157	13,087	10,308	9,866	9,248	58%
	SUW	7,291	5,914	5,619	5,312	7,414	10,133	10,158	11,667	11,373	10,655	56%
Other operating expenses	BIA	577	268	487	806	1,322	1,653	3,830	4,300	5,310	22,422	821%
	LOM	5,304	2,210	306	563	580	5,998	1,861	3,350	1,327	11,295	−75%
	SUW	3,111	3,291	2,713	1,732	2,400	5,299	2,040	4,353	1,481	1,877	−52%
Financial revenue	BIA	41	346	42	36	36	233	59	261	135	330	233%
	LOM	42	125	149	147	89	65	20	401	640	136	1,423%
	SUW	90	35	75	41	39	17	12	176	173	152	93%
Financial costs (interest)	BIA	1,953	1,644	1,760	1,770	2,676	2,564	3,244	5,796	12,571	13,135	544%
	LOM	528	266	149	95	33	50	2	1	12	105	−98%
	SUW	1,071	838	704	697	663	562	361	733	173	152	−84%
Profit (loss) on sales	BIA	13,459	−15,141	−18,672	−28,841	−38,994	−46,107	−67,867	−52,897	−46,004	−52,885	−442%
	LOM	1,886	2,134	148	−11,826	−16,664	−6,776	−19,209	−17,840	−16,889	−28,273	−995%
	SUW	−4,063	−2,273	−3,632	−5,726	−6,385	−8,299	−10,655	−10,292	−11,690	−16,296	188%
Profit on operating activities	BIA	1,934	−15,141	−18,672	−9,139	−11,778	−1,068	−7,257	−12,204	−9,200	−34,751	−576%
	LOM	3,572	2,134	148	−11,826	−16,664	−6,776	−19,209	−17,840	−5,011	−26,919	−240%
	SUW	1,264	−2,273	−3,632	1,257	1,262	1,578	−10,655	−10,292	918	887	−27%
Gross result	BIA	22	18	−4,650	−10,873	−14,418	−3,399	−10,442	−17,739	−21,636	−47,556	−99,524%
	LOM	3,085	8,931	11,576	−3,019	−8,447	941	534	244	−4,383	−26,888	−242%
	SUW	283	1,019	857	601	638	1,033	534	607	246	104	−13%
Net result	BIA	22	18	−4,650	−10,873	−14,418	−3,399	−10,442	−17,739	−21,636	−47,556	−99,524%
	LOM	2,926	8,800	10,674	−3,063	−8,447	907	503	209	−4,515	−26,888	−254%
	SUW	271	1,015	856	571	130	1,029	2,060	603	136	104	−50%

**Table 3:** DEA efficiency scores for different models (CCR, BCC) stratified by hospital and period with 95% CI.

	Means (95% CI)							
	Pre-pandemic (2015–2019)		Pandemic (2020–2021)		Post-pandemic (2022–2024)		Overall (2015–2024)	
Hospital	CCR	BCC	CCR	BCC	CCR	BCC	CCR	BCC
Operational set								
BIA	0.8540 (0.8040–0.8980)	0.9200 (0.8700–0.9520)	0.7460 (0.7040–0.7830)	0.8000 (0.7630–0.8220)	0.8580 (0.7810–0.9460)	0.9160 (0.8410–0.9580)	0.8340 (0.7777–0.8890)	0.8950 (0.8400–0.9280)
LOM	0.6070 (0.5710–0.6430)	0.8950 (0.8640–0.9160)	0.3990 (0.3710–0.4100)	0.8520 (0.8230–0.8720)	0.6020 (0.5650–0.6340)	0.8490 (0.8280–0.8620)	0.5630 (0.5290–0.5930)	0.8730 (0.8450–0.8910)
SUW	0.9090 (0.8330–0.9890)	0.9550 (0.8930–0.9980)	0.8100 (0.7310–0.8700)	0.9600 (0.9060–0.9980)	0.8300 (0.7580–0.9130)	0.9600 (0.9060–0.9980)	0.8650 (0.7900–0.9940)	0.9570 (0.8990–0.9980)
Financial set								
BIA	0.8900 (0.8610–0.9070)	0.9780 (0.9270–0.9960)	0.7850 (0.7660–0.7980)	0.8360 (0.8210–0.8430)	0.9280 (0.8630–0.9590)	0.9560 (0.9000–0.9720)	0.8800 (0.8420–0.9010)	0.9430 (0.8980–0.9590)
LOM	0.9420 (0.9190–0.9570)	0.9500 (0.9340–0.9590)	0.9600 (0.9470–0.9700)	0.9640 (0.9550–0.9720)	0.9400 (0.9190–0.9580)	0.9640 (0.9360- 0.9760)	0.9460 (0.9240–0.9600)	0.9570 (0.9390–0.9670)
SUW	0.9590 (0.9050–0.9950)	0.9730 (0.9170–0.9960)	0.9510 (0.9250–0.9670)	0.9880 (0.9680–0.9980)	0.9490 (0.9010–0.9790)	0.9810 (0.9320–0.9990)	0.9540 (0.9070–0.9850)	0.9780 (0.9310–0.9970)

CCR—DEA model under constant returns to scale reporting total technical efficiency.

BCC—DEA model under variable returns to scale reporting pure technical efficiency.

DEA results were stratified into three period blocks: pre-pandemic (2015–2019), pandemic (2020–2021), and post-pandemic (2022–2024).

CI, confidence interval.

### Operational efficiency (CCR, BCC): management of beds and staff

Over the decade analysed, average operational TE_CCR scores were 0.8340 for BIA, 0.5630 for LOM and 0.8650 for SUW, whereas average PTE_BCC scores were 0.8950, 0.8730 and 0.9570, respectively (Table 3). In an input-oriented interpretation, this means that BIA could reduce the number of beds and staff by 16.6% while maintaining the same number of hospitalisations and surgical procedures. A PTE_BCC score of 0.8950 indicates that 10.5% of the gap to the efficiency frontier results from organisational factors, and 6.1% from a mismatch between the hospital's scale of operation and regional needs. Most of BIA's resources were therefore used relatively efficiently, and the remaining inefficiency stems from scale rather than from suboptimal management of clinical staff.

Over 2015–2024, LOM achieved an average operational TE_CCR score of 0.5630, meaning that its operational efficiency was 56.3% and that the hospital could, in theory, reduce the number of beds and staff by 43.7% while maintaining the same numbers of hospitalisations and surgical procedures. A PTE_BCC score of 0.8730 implies that 12.7% of inefficiency can be attributed to the hospital's internal organisation of personnel, whereas 31.0% of inefficiency is due to a misalignment between the size and scale of the hospital and the needs of the region. DEA therefore points to a substantial surplus of resources in LOM throughout the period.

SUW maintained the highest operational PTE_BCC throughout, with an average BCC score of 0.9570 and confidence intervals frequently including 1.00, and a TE_CCR score of 0.8650. This translates into a potential reduction in inputs of 13.5% while keeping the number of hospitalisations and surgical procedures unchanged. The remaining 4.3% gap to full (100%) efficiency is due to pure technical inefficiency of the hospital. SUW appears to manage beds and staff efficiently, and the potential for improvement relates mainly to adjusting scale rather than to a fundamental overhaul of clinical workforce management.

The operational DEA results are consistent with the descriptive indicators (Table 1). SUW increased the number of patients and throughput while slightly reducing the number of beds and shortening LOS, which corresponds to high organisational efficiency (PTE_BCC) and a moderate scale-related gap in TE_CCR. BIA combined a marked reduction in bed numbers with a large increase in hospitalisations and throughput, and the efficiency analysis confirmed no major departure from the technical efficiency frontier despite substantial changes in key organisational components. By contrast, LOM operated with a large reserve of potential efficiency relative to the organisational resources at its disposal, which is reflected in its low TE_CCR score.

### Financial efficiency (CCR, BCC): cost management

In 2015–2024, financial efficiency was uniformly higher and more stable than operational efficiency across periods, and the differences between TE_CCR and PTE_BCC were smaller than in the operational model (Figures 3, 4). Over the decade, average financial TE_CCR scores were 0.8800 in BIA, 0.9460 in LOM and 0.9540 in SUW, whereas average PTE_BCC scores were 0.9430, 0.9570 and 0.9780, respectively (Table 3).

For BIA, the decade-average TE_CCR of 0.8800 means that, holding revenues and activity (number of hospitalisations and surgical procedures) constant, total operating costs could be reduced by about 12%. Over the same period, PTE_BCC was 0.9430, implying that 5.7 percentage points of inefficiency are related to financial management and cost structure, while 6.3 percentage points arise from a suboptimal scale of operations. The cost structure therefore appears reasonably aligned with the volume of services, and the scope for further cost optimisation without affecting the number of hospitalisations and surgical procedures is limited.

In 2015–2024, LOM operated close to the financial efficiency frontier (TE_CCR = 0.9460, PTE_BCC = 0.9570). The potential reduction in costs at unchanged numbers of hospitalisations and surgical procedures is 5.4%, of which 4.3 percentage points of inefficiency can be attributed to the organisation of hospital finances. Despite this, the hospital's net result deteriorated from a surplus of approximately PLN 2.9 million in 2015 to a deficit of about PLN 26.9 million in 2024 (Table 2).

SUW displayed the most stable financial profile and achieved the highest financial efficiency (TE_CCR = 0.9540, PTE_BCC = 0.9780), with confidence intervals often including 1.00 (Table 3). The hospital converted rising personnel and material costs into revenues and output with minimal losses. Scale efficiency remained close to 0.97, with propagated confidence intervals overlapping 1.00 in multiple years (Figure 6).

### Cross-domain summary (SE): scale efficiency and returns to scale

Scale efficiency (SE) trajectories diverged between domains. In the operational model, BIA was characterised by DRS and SE below 1.00 for most of the decade. This indicates that the hospital operated at a scale that was too large relative to its output: additional beds and staff generated less-than-proportional increases in hospitalisations and procedures. This is consistent with the DEA results, which suggest that part of the gap between TE_CCR and PTE_BCC reflects a scale mismatch rather than pure technical inefficiency. Gradual reductions in bed numbers combined with rising activity improved SE in the post-pandemic period and resulted in BIA being classified as operating under CRS in 2024. This can be interpreted as convergence towards an approximately optimal size: further expansion would be unlikely to produce proportional gains in output, whereas small reductions in scale could be offset by process reorganisation. In the financial model, SE remained high, suggesting that scale-related issues in BIA primarily concern the use of clinical resources rather than the relationship between costs and revenues.

In LOM, SE in the operational model was the lowest among the hospitals, and the hospital was classified as DRS in every year. In combination with relatively high PTE_BCC scores, this pattern indicates structural oversizing: processes are organised reasonably well, but the scale of operations is persistently too large for the number of patients treated. Reductions in bed numbers did not keep pace with the decline in hospitalisations, which resulted in sustained low bed occupancy and a small number of patients per bed. From a DEA perspective, additional units of resources in LOM act more as a buffer than as a genuine potential for higher production, and adding resources would not translate into proportional increases in hospitalisations and surgical procedures. Even the period when LOM was transformed into a COVID-19 dedicated hospital did not change its RTS classification, indicating that excess capacity is structural and driven more by long-term organisational characteristics (profile, ward network, role in the system) than by short-term demand fluctuations. In the financial model, SE was close to 1.00, confirming that oversizing mainly concerns physical infrastructure and clinical staff rather than the cost–revenue relationship.

Over the decade, SUW was distinguished by the highest SE in the operational model and the most favourable RTS profile. Before the pandemic, the hospital was most often classified as CRS, meaning that it operated close to the optimal scale and that proportional increases in beds and staff could generate roughly proportional increases in output. During the COVID-19 period 2020–2021, SUW shifted to DRS, reflecting the forced maintenance of additional reserve capacity in beds and staff alongside constrained elective activity. The decline in SE was therefore temporary and related to the need to secure uncertain pandemic demand. After 2022, SUW gradually returned to a profile close to CRS and was classified as CRS again in 2024. In combination with high PTE_BCC, this classification indicates an optimal match between the hospital's scale and the needs of its catchment area, and any further expansion or downsizing should be undertaken cautiously to avoid shifting the hospital into a persistent DRS regime. In the financial model, SE was high, indicating that SUW balances costs and revenues well at its current scale of operations.

### Second-stage (exploratory) truncated regression

As an exploratory robustness check, truncated regressions were estimated with inefficiency (1—BCC) as the dependent variable. In the operational model, longer average LOS was positively associated with inefficiency (estimate 0.0283, *p* = 0.0002), whereas bed occupancy and the share of contracted physicians were not statistically significant (Table 4). An increase in LOS by one day corresponded, on average, to a 0.0283-point rise in operational inefficiency, holding other variables constant. Thus, the longer patients stay in hospital and the longer beds remain occupied, the greater the proportion of resources that cannot be reused, making it substantially more difficult to maintain high operational efficiency at a fixed number of staff and beds. This finding is consistent with the DEA results, according to which hospitals with shorter LOS were closer to the efficiency frontier, whereas hospitals with prolonged hospitalisations (LOM and, in some years, BIA) displayed higher inefficiency. The share of physicians employed under civil-law contracts had a small, positive, but statistically non-significant coefficient. In practice, this indicates that in the period analysed, the form of physician employment was not a strong, independent determinant of operational inefficiency; contract-based employment itself was not a key driver of how efficiently beds and staff were used.

**Table 4:** Truncated regression of operational inefficiency.

Coefficient	Estimate	Standard Error	*p*-value
Intercept	−0.0364	0.1352	0.7877
Bed occupancy	−0.0016	0.0015	0.2860
LOS (average length of stay)	0.0283	0.0076	**0**.**0002**
Physicians' contract share (%)	0.0011	0.0007	0.1447
COVID (0/1)	0.0335	0.0297	0.2589
Post-COVID (0/1)	0.0247	0.0221	0.2620

VRS, variable returns to scale; LOS, average length of stay. Model fitted with truncated regression.

Bold values indicate statistical significance at *p* < 0.05.

In the financial model, the share of contracted physicians (estimate 0.0031, *p* = 0.0383) and the share of surgical cases among inpatients (estimate 0.4719, *p* = 0.0351) were positively associated with financial inefficiency. A 10-percentage-point increase in the share of contracted physicians (for example, from 40% to 50%) was associated, on average, with a 0.031 increase in financial inefficiency (10 × 0.0031). In practice, hospitals that rely more heavily on contract-based employment tend to find it more difficult to keep total costs (especially wages) at a level commensurate with their output and revenues. The share of surgical cases among inpatients, expressed as a proportion between 0 and 1, was also statistically significant. An increase in this share by 0.1 (10 percentage points) was associated with a 0.047 increase in financial inefficiency, holding other variables constant. These results suggest that a high surgical specialisation raises cost burdens more than revenues, thereby worsening financial efficiency as measured by DEA. Bed occupancy showed a positive but borderline association (*p* = 0.065), while LOS was not significant in the financial model (Table 5). Period covariates showed a marginally positive association for 2020–2021 (*p* ≈ 0.092) and no significant difference for 2022–2024 vs. 2015–2019.

**Table 5:** Truncated regression of financial inefficiency.

Coefficient	Estimate	Standard Error	*p*-value
Intercept	−0.7182	0.3617	**0**.**0471**
Beds	−0.0001	0.0003	0.7226
Bed occupancy	0.0052	0.0028	0.0647
LOS (average length of stay)	0.0151	0.0209	0.4710
Physicians' contract share (%)	0.0031	0.0015	**0**.**0383**
Surgery share	0.4719	0.2240	**0**.**0351**
COVID (0/1)	0.0810	0.0480	0.0918
Post-COVID (0/1)	−0.0329	0.0523	0.5294

VRS, variable returns to scale; LOS, average length of stay. Model fitted with truncated regression.

Bold values indicate statistical significance at *p* < 0.05.

The second-stage models were estimated on a small sample, so these results should be interpreted as descriptive rather than causal. At the same time, they are consistent with the main DEA findings, indicating that differences in efficiency are indeed related to the intensity of bed use (LOS) and to cost structure (contract-based employment, share of surgical cases), rather than reflecting random variation alone.

## Discussion

This ten-year analysis of three provincial hospitals in Podlaskie Voivodeship (2015–2024) reveals a distinct shock-and-recovery pattern. Operational efficiency declined sharply in 2020–2021 and partially rebounded after 2022, whereas financial efficiency remained comparatively stable and closer to the frontier (31). Bootstrap percentile 95% confidence intervals for TE_CCR and PTE_BCC confirm that several estimates differed from unity (Table 3; Figures 1–6), indicating tangible deviations from annual frontiers of relative efficiency (32, 33). Taken together, these trajectories support H1 (pandemic-related decline with partial post-2022 recovery) and H3 (between-hospital heterogeneity), while the frequent pattern of PTE_BCC ≈ 1.00 with TE_CCR < 1.00 is consistent with H2, pointing to scale-related rather than purely technical shortfalls. The weak alignment between operational and financial scale efficiency described below is in line with H4, suggesting partly independent adjustment paths in the two domains.

### Efficiency and scale—two separate dimensions

Differences between CCR and BCC results are interpreted through scale efficiency (SE = TE_CCR/PTE_BCC) (34). In several years PTE_BCC was close to 1.00 while TE_CCR remained below 1.00, suggesting that hospitals operated efficiently in technical terms but at a suboptimal scale (35). This pattern was most pronounced in the operational model during 2020–2021 and persisted, to a lesser extent, after 2022, when patient throughput and bed occupancy remained unstable. The RTS diagnostics corroborate this interpretation: DRS predominated across hospitals, with occasional CRS and no material IRS (Table 3A). Under such conditions, expanding resources does not proportionally increase outputs; hospitals functioned with some degree of excess capacity relative to demand—an observation particularly clear in Lomza (LOM) during 2020–2021, when part of the infrastructure remained underused (36).

**Table 3A:** Returns-to-scale (RTS) classification by period and hospital in the operational model (2015–2024).

Hospital	Period	CRS (years)	DRS (years)	IRS (years)
BIA	Pre-COVID	0	5	0
	COVID	0	2	0
	Post-COVID	1	2	0
LOM	Pre-COVID	0	5	0
	COVID	0	2	0
	Post-COVID	0	3	0
SUW	Pre-COVID	4	1	0
	COVID	0	2	0
	Post-COVID	1	2	0

CRS, constant returns to scale; DRS, decreasing returns to scale; IRS, increasing returns to scale. Counts denote the number of years in each category per period.

### Divergent trajectories within a unified system

Despite operating under the same contracting and regulatory framework, the three hospitals followed distinct efficiency paths (37). Suwalki (SUW) consistently performed near the efficiency frontier in both domains, reflecting a close alignment between operating scale and service volume. Bialystok (BIA) showed greater year-to-year variability but demonstrated clear operational improvement after 2022, coinciding with higher bed turnover and more balanced resource use. Lomza (LOM) experienced the steepest operational decline in 2020–2021, corresponding with its temporary conversion into a COVID-19-dedicated facility (38). This reconfiguration produced a marked mismatch between resource scale and actual patient flow, resulting in strong DRS. After 2022, efficiency improved but did not return to pre-pandemic levels, indicating persistent constraints in capacity utilisation.

### A borderland region—balancing efficiency and readiness

Podlaskie is a sparsely populated, peripheral region situated on the eastern border of the European Union and NATO. Its hospital network therefore serves a dual role: routine service provision and health-security infrastructure (39, 40). Within this context, the DEA findings take on strategic relevance. The predominance of DRS should not be read simply as wastefulness; it can also be interpreted as structural reserve capacity that allows hospitals to absorb sudden demand surges, such as those observed during the pandemic or in potential migration and military emergencies (41, 42). In LOM, the pronounced DRS in 2020–2021 is consistent with its designated one-disease status rather than deliberate reserve keeping. The data do not show that this reserve was fully utilised; its usability was constrained by system-level decisions and the hospital's assigned role. Hence, the observed inefficiency mainly reflects a temporary scale misalignment imposed by specialisation rather than intrinsic managerial under-performance (43).

### Operations and finances—different rhythms of adjustment

We did not observe a clear year-by-year alignment between operational and financial scale efficiency, supporting H4 and suggesting that organisational and financial adjustments unfold at different tempos. Operational efficiency responds rapidly to changes in patient flow, bed occupancy, and case-mix, whereas financial performance is cushioned by contractual mechanisms and delayed cost recognition (44). Consequently, short-term activity swings do not automatically translate into financial results—a typical feature of publicly funded hospitals with complex service portfolios (45, 46). This decoupling helps to explain why financial efficiency remained near the frontier even when operational efficiency temporarily deteriorated.

### Sources of inefficiency—insights from the second stage

In exploratory truncated regressions (dependent variable = 1—PTE_BCC), several contextual variables were associated with inefficiency. In the operational model, LOS was positively associated with inefficiency, consistent with reduced turnover and less intensive use of existing capacity. In the financial model, a higher share of contracted physicians and a greater proportion of surgical cases were linked to higher inefficiency, in line with cost pressures from contract employment and procedure-intensive case-mixes. This pattern may indicate that flexible, activity-dependent forms of employment and high surgical volumes generate additional costs that are not fully recognised or rewarded by the current payment arrangements of the National Health Fund. These results are descriptive and based on a small panel but support the interpretation that inefficiency arises not only from managerial shortcomings but also from structural and organisational conditions—particularly in regions with limited budgets and dispersed demand.

### Efficiency vs. resilience

The observed trajectories distinguish efficiency, defined as the input–output relation at a given point in time, from resilience, understood here as the system's ability to move back towards the efficiency frontier after a disturbance (47, 48). The post-2021 recovery of TE_CCR, PTE_BCC, and SE exemplifies descriptive resilience rather than modelled causality. We do not infer specific mechanisms; instead, we document that hospitals were able to regain much of their pre-pandemic relative position. In a borderland setting, where demographic and geopolitical uncertainties persist, maintaining such adaptive capacity becomes an important component of regional health security—even if it implies operating slightly below the efficiency frontier in normal periods (49).

### Practical interpretation and limitations

Within the DEA logic, instances of PTE_BCC ≈ 1.00 with TE_CCR < 1.00 identify situations where scale adjustment—rather than process improvement—is the main lever of efficiency (50). For peripheral regions like Podlaskie, the practical challenge is to track whether deviations from the frontier arise primarily from scale or from pure technical factors and how this balance changes over time (51). In this sense, routine, period-stratified reporting of operational and financial DEA indicators may support transparent monitoring of hospital performance without prescribing specific policies. Key methodological constraints—including the small sample (3 hospitals × 10 years), deterministic DEA, lack of risk adjustment, and the exploratory nature of second-stage models—are discussed in the Limitations section and should be borne in mind when interpreting these findings (52).

## Conclusions

This ten-year study of three provincial hospitals in Podlaskie Voivodeship (2015–2024) shows that hospital efficiency is a dynamic property shaped by both operational scale and external shocks. A clear pandemic-related dip in 2020–2021, followed by partial convergence after 2022, reflects changing positions relative to the annual efficiency frontier rather than a single, fixed level of performance. Most deviations from the frontier arose from scale-related factors, while the three hospitals followed distinct trajectories despite a uniform regulatory environment.

Operational and financial domains evolved only partly in parallel. Operational efficiency was more sensitive to changes in patient flows and bed use, whereas financial efficiency remained close to the frontier across periods. However, this high financial efficiency did not preclude the emergence of operating deficits, particularly in LOM, illustrating that proximity to the financial frontier can coexist with deteriorating balance sheets under budget-based contracts. In a border-region context on NATO's eastern flank, these patterns suggest that hospitals may systematically operate with some reserve capacity, visible as DRS and SE below 1.00 in the operational domain. Rather than treating this solely as inefficiency, DEA-based, period-stratified monitoring of operational and financial indicators can document how the balance between efficiency and preparedness evolves over time, supporting transparent reporting on hospital performance without prescribing specific structural changes. From a practical perspective, integrating such DEA-based monitoring into routine regional planning and into the design of National Health Fund contracts could help align incentives so that they recognise both efficient use of resources and the need for maintained reserve capacity in strategically sensitive border regions.

## Limitations

This study has several limitations. First, the analysis covers only three provincial hospitals observed over ten years (30 DMUs). With such a small peer set, DEA frontiers are sensitive to individual observations, and results cannot be generaliSed beyond similar regional hospitals. Second, DEA is a deterministic, non-parametric method that attributes all deviations from the frontier to inefficiency. Random noise, measurement error in routinely collected data, and unobserved shocks cannot be separated from genuine managerial or structural gaps.

Third, we did not apply formal risk adjustment for case-mix or clinical complexity, and the input–output sets do not include quality or outcome indicators. The operational model focuses on inpatient throughput and staffing; the financial model captures costs, revenues, and activity levels in nominal PLN. Benchmarking is strictly within-year, which reduces the impact of price changes, but period summaries across 2015–2024 should be read as descriptive rather than as formal trends. Fourth, bootstrap uncertainty was quantified only for TE_CCR and PTE_BCC; scale efficiency was derived as their ratio without separate confidence intervals, so inferences about SE rely on its components.

Fifth, during the pandemic LOM temporarily served as a COVID-19-dedicated hospital, which reduced its comparability to BIA and SUW in 2020–2021. The resulting operational inefficiency largely reflects this special role and should not be interpreted as a stable characteristic of the hospital. Sixth, the second-stage truncated regressions are exploratory: they are based on a small sample, include a limited set of covariates, and cannot support causal inference. Coefficients should therefore be viewed as hypothesis-generating rather than explanatory.

Finally, our interpretation of crisis-time and borderland patterns is informed by international literature on hospital efficiency and resilience, mostly from non-border regions. While this helps situate the findings, external evidence may not fully capture the specific geopolitical and demographic context of Podlaskie, and extrapolation to other settings should be cautious.

## Data Availability

The original contributions presented in the study are included in the article/Supplementary Material, further inquiries can be directed to the corresponding author.
